# Urinary calculi successfully expelled in a patient through traditional Chinese exercise: A case report

**DOI:** 10.1097/MD.0000000000040641

**Published:** 2024-11-22

**Authors:** Chengheng You, Jing Xian Li, Guanwu Li, Rongliang Dun, Min Fang, Qingguang Zhu

**Affiliations:** a Yueyang Hospital of Integrated Traditional Chinese and Western Medicine, Shanghai University of Traditional Chinese Medicine, Shanghai, China; b School of Human Kinetics, Faculty of Health Sciences, University of Ottawa, Ottawa, ON, Canada; c Radiology Department, Yueyang Hospital of Integrated Traditional Chinese and Western Medicine, Shanghai University of Traditional Chinese Medicine, Shanghai, China; d Urology Department, Yueyang Hospital of Integrated Traditional Chinese and Western Medicine, Shanghai University of Traditional Chinese Medicine, Shanghai, China; e Research Institute of Traditional Chinese Exercise, Shanghai Institute of Traditional Chinese Medicine, Shanghai, China.

**Keywords:** case report, non-surgical methods, stone expulsion, traditional Chinese exercises, urinary calculi

## Abstract

**Rationale:**

Urinary calculi are hard mineral deposits that typically require medication or surgery, such as lithotripsy. This case report presents traditional Chinese exercises (TCEs) as a potential alternative for stone expulsion.

**Patient concerns:**

A 41-year-old male with no history of urinary tract stones, experienced sudden severe lower back and abdominal pain accompanied by nausea and vomiting.

**Diagnosis:**

Computed tomography scan revealed a small calculus at the distal end of the left ureter (within the bladder wall), approximately 2 mm in size, with mild hydronephrosis in the ureter and renal pelvis.

**Interventions:**

The patient was initially prescribed medication for pain relief and was advised to engage in TCEs.

**Outcomes:**

Follow-up computed tomography scan after the exercise regimen showed complete expulsion and disappearance of the urinary calculi. The patient reported significant improvement in physical and mental health with no recurrence of calculi observed in subsequent checkups.

**Lessons:**

This case suggests that TCEs may facilitate the expulsion of small urinary calculi, offering a noninvasive treatment option. Further research is needed to confirm the therapeutic effects of TCEs on urinary calculi and to explore its potential mechanisms.

## 1. Introduction

Urinary calculi are a prevalent urological condition globally, with a prevalence exceeding 5%,^[1]^ capable of causing excruciating pain for patients.^[2]^ Traditional treatments mainly include pharmacotherapy, extracorporeal shock wave lithotripsy, and endoscopic surgery. However, non-surgical methods may be more appropriate for patients with small urinary calculi (diameter <5 mm).^[3]^ Research indicates that moderate exercise and physical activity can reduce the incidence of urinary tract stones,^[4–6]^ which suggests potential benefits of non-surgical methods. Traditional Chinese exercises (TCEs) are an ancient form of physical practice originating from China, encompassing movements such as Tai Chi and the Eight Pieces of Brocade, aimed at promoting health and well-being. TCEs have been proven to have adjunctive therapeutic effects for many ailments, such as hypertension, cardiovascular diseases, and psychological disorders.^[7–10]^ This case report aims to explore the application of TCEs in patients with small urinary calculi.

## 2. Case report

**Figure 1.**
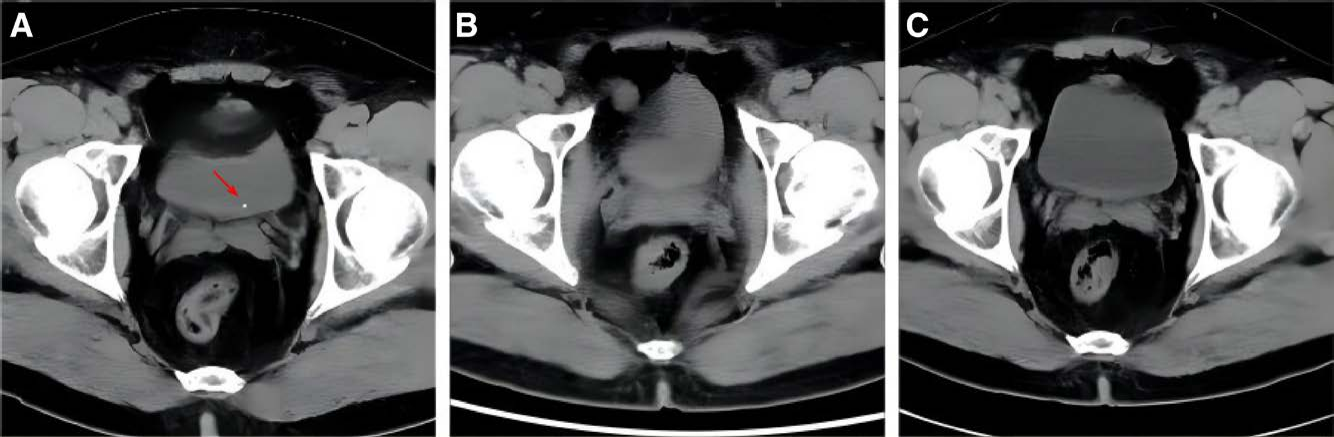
CT of urinary tract imaging on March 27, 2022: a small calculus at the distal end of the left ureter (within the bladder wall), approximately 2 mm in size, with mild hydronephrosis in the ureter and renal pelvis. (A) CT of urinary tract imaging on May 19, 2022: no urinary tract stones found. (B) CT of urinary tract imaging on April 14, 2023: no urinary tract stones found. (C) Detailed CT images can be found in the supplementary materials. CT = computed tomography.

The patient, a 41-year-old male with no history of urinary tract stones, experienced sudden severe lower back and abdominal pain accompanied by nausea and vomiting on March 27, 2022.He was admitted to the emergency department, where a computed tomography (CT) scan revealed a small calculus at the distal end of the left ureter (within the bladder wall), approximately 2 mm in size, with mild hydronephrosis in the ureter and renal pelvis (Fig. 1A). The patient was administered prednisone 60 mg intramuscularly, atropine 1 mg intramuscularly, nitrous oxide 250 mL with antipyrine 120 mg IV infusion, and celecoxib 0.2 g PRN by mouth, which gradually alleviated the lower back and abdominal pain, as well as nausea and vomiting. After the emergency treatment, the patient consulted a urologist about daily exercise methods for treating ureteral calculi. The urologist advised the patient to learn TCEs and maintain a consistent exercise regimen.

The patient was placed under home quarantine following a Coronavirus disease outbreak in Shanghai on March 28, 2022. After experiencing a noticeable improvement in symptoms, The patient started following a series of instructional videos on MOOC titled “TECs for Health” on April 1, 2022, without taking any medication or undergoing other treatments. The patient adhered to the doctor’s recommendation, engaging in two 20-minute exercise sessions daily, managing pain as necessary, without undergoing medication or other treatments. Between May 3 and May 17, the patient experienced occasional stinging pain during stone expulsion, particularly on May 16 and 17 when he felt pain in the urethra and noticed mild hematuria. Consequently, the patient underwent another CT scan on May 19, which revealed the disappearance of the urinary calculi (Fig. 1B).

After the initial period of consistent TCE practice, the patient demonstrated a remarkable recovery trajectory. Posttreatment, he reported a sustained amelioration in both his physical and mental health, with no discomfort noted subsequent to his exercises. A follow-up CT scan conducted on April 14, 2023, confirmed the complete absence of calculi in the kidneys and ureters (Fig. 1C), validating the effectiveness of TCEs in stone expulsion. Beyond the primary outcome, the patient experienced ancillary benefits: his lower back pain was mitigated, and his insomnia was significantly reduced. These improvements prompted us to recommend ongoing TCE practice as a preventive measure against future stone formation. Our continued follow-up with the patient through February 2024 revealed no return of urinary stone symptoms, underscoring the enduring impact of TCEs on his overall health.

## 3. Traditional Chinese exercises: Movements and techniques

### 3.1. Action 1 (Fig. 2)

*Movement*: Place both elbow joints flexed in front of the chest, bilateral flexion (2 and 3) and extension (4 and 5) of the knee joint.

**Figure 2.**
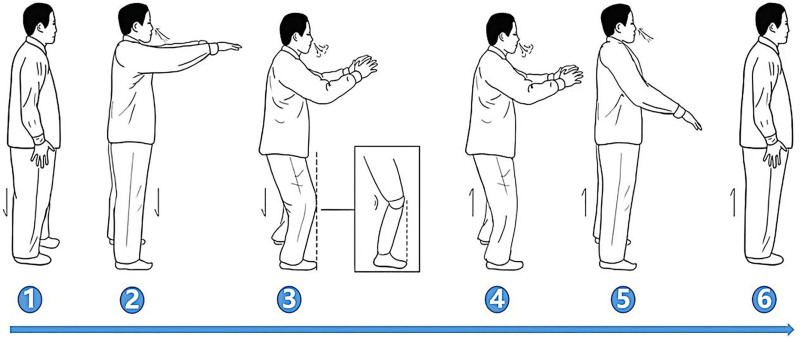
Action 1.

*Breathing rhythm*: Inhalation (2 and 3) and exhalation (4 and 5).

*Meditation*: Staring at the floor approximately 3 m ahead (2 and 3), focus on the position of the knee joint (4 and 5).

*Benefits*: The chest-expanding movement coupled with knee flexion and extension creates changes in thoracoabdominal pressure. This pressure variation is beneficial for promoting the expulsion of urinary calculi by facilitating their movement through the urinary tract.

### 3.2. Action 2 (Fig. 3)

*Movement*: Both heels off the ground, toes grasping, both arms extended forward (2 and 3); both heels quickly drop to the ground with both upper limbs flexed backwards (4 and 5).

**Figure 3.**
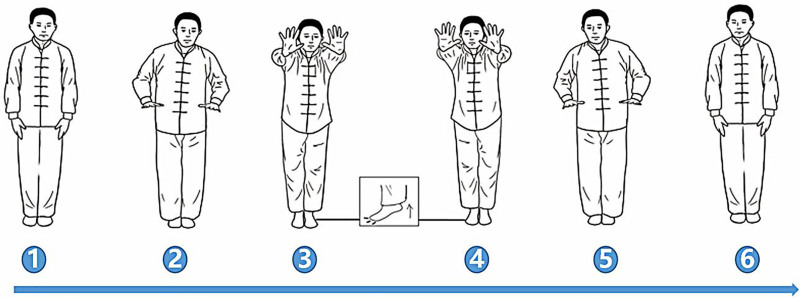
Action 2.

*Breathing rhythm*: Inhalation (2 and 3) and exhalation (4 and 5).

*Meditation*: Staring at the floor 3 m ahead (2 and 3), focus on the position of the knee joint (4 and 5).

*Benefits*: The upward and downward movement of the heels generates a gentle vibration throughout the body. This vibration is advantageous for the expulsion of urinary calculi by aiding in their movement and potentially dislodging them from the urinary tract (see Video S1, Supplemental Digital Content, http://links.lww.com/MD/O22 which demonstrates the movements of the 2 TCEs mentioned above).

## 4. Discussion

In this case, the patient successfully expelled urinary calculi and alleviated pain by consistently engaging in TCEs. These exercises can increase blood circulation,^[11]^ metabolism,^[12]^ reduce anxiety levels,^[13]^ strengthen immunity, and significantly improve chronic diseases and suboptimal health conditions.^[14]^

TCEs, as a non-pharmacological treatment, have significant potential for patients with urinary calculi. This case report suggests that these exercises may facilitate the expulsion of urinary calculi and alleviate pain. These TCEs can be considered for patients with urinary calculi under the supervision of a doctor. However, further research and case studies are needed to confirm the therapeutic effects of TCEs on patients with urinary calculi.

TCEs may not be suitable for all patients with urinary calculi, especially for those whose physical limitations may prevent them from performing the exercises safely. Pharmacotherapy, extracorporeal shock wave lithotripsy, or minimally invasive surgery may still be required for patients with larger stones or unfavorable locations for expulsion. Patients must consult a medical professional before initiating TCEs to understand their specific condition and develop an appropriate treatment plan.

Furthermore, patients should practice TCEs under professional supervision to ensure accurate movements and avoid harm from improper exercise. Patients should closely monitor their physical condition when engaging in the exercise routine and promptly seek medical attention if any discomfort arises.

## 5. Conclusion

In summary, this case report demonstrates the positive therapeutic effects of TCEs for patients with urinary calculi and provides new insights into non-surgical treatments for this condition, such as the potential role of TCEs in facilitating the natural expulsion of small stones and improving overall patient well-being.

Based on the success of this case, we propose the following guidelines for implementing TCEs in clinical practice:

1. *Initial assessment*: Conduct a thorough evaluation of the patient’s condition before initiating TCEs to ensure the exercises are suitable for individual needs.
2. *Professional guidance*: Recommend that patients perform TCEs under the guidance of a qualified instructor to ensure proper execution of the movements.
3. *Gradual intensity*: Increase the frequency and intensity of TCEs gradually based on the patient’s adaptability.
4. *Holistic treatment*: Consider TCEs as part of a comprehensive treatment plan, complementing traditional medical therapy.
5. *Ongoing monitoring*: Regularly track patient progress and adjust the treatment plan as necessary.

Future research should further explore the mechanisms of TCEs in treating urinary calculi to provide more guidance for clinical practice.

## Acknowledgments

We would like to express our gratitude to the patient for granting permission to use their clinical data in this paper and for the publication of this research.

## Author contributions

**Conceptualization:** Min Fang, Qingguang Zhu.

**Investigation:** Rongliang Dun.

**Supervision:** Jing Xian Li, Min Fang, Qingguang Zhu.

**Visualization:** Guanwu Li.

**Writing – original draft:** Chengheng You.

**Writing – review & editing:** Chengheng You.

## Supplementary Material


